# Erythrodermic crusted scabies in an immunocompetent three-month-old infant with marked systemic inflammation^؆^

**DOI:** 10.1016/j.abd.2026.501389

**Published:** 2026-06-18

**Authors:** Márcio Fellipe Menezes Viana, Renata Ferreira Magalhães, Andréa Fernandes Eloy da Costa França

**Affiliations:** a Department of Internal Medicine, Division of Dermatology, Universidade Estadual de Campinas, Campinas, SP, Brazil; b Department of Internal Medicine, Division of Dermatology, Universidade Estadual de Campinas, Campinas, SP, Brazil

Dear Editor,

Crusted scabies is a hyperinfestation by Sarcoptes scabiei characterized by extensive hyperkeratosis, a very high mite burden, and extraordinary contagiousness. It is classically linked to immunosuppression or reduced ability to scratch, and remains exceptional in otherwise healthy infants.^1^, ^2^ We report an immunocompetent infant with erythroderma and marked systemic inflammation in whom bedside dermoscopy expedited diagnosis and helped control a household outbreak.

A previously healthy 3-month-old girl was admitted with a one-month history of rapidly progressive pruritic papulo-edematous eruption evolving to generalized erythroderma. She was afebrile and hemodynamically stable but markedly irritable. Cutaneous examination showed diffuse erythema with widespread fine scaling and thick adherent hyperkeratotic crusts, most prominent on the dorsal trunk and proximal upper limbs (Fig. 1). There was no mucosal involvement. The family reported preserved weight gain and no history of prematurity, malnutrition, recurrent infections, or exposure to immunosuppressive medications. Laboratory evaluation showed leukocytosis (32,500 µL) with marked eosinophilia (7,700 µL) and lymphocytosis (14,400 µL), without left shift, and elevated C-reactive protein (56 mg/L). No clinical or laboratory evidence of secondary bacterial infection was identified. Serum immunoglobulin levels were within the normal range for age, and there were no clinical features suggestive of primary immunodeficiency.

**Fig. 1:**
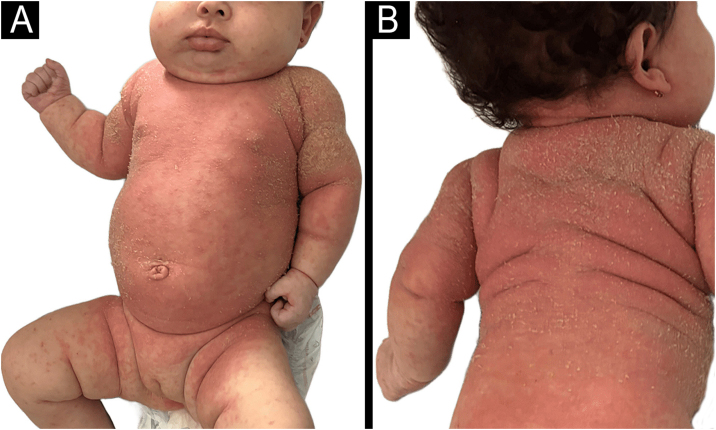
Clinical presentation. (A) Generalized erythroderma with diffuse fine scaling and thick adherent hyperkeratotic crusts involving the trunk and upper limbs. (B) Posterior view highlighting confluent erythema and widespread crusting on the upper back.

Given the combination of erythroderma, hyperkeratotic crusts, and intense pruritus, scabies was suspected. Dermoscopy performed at the bedside revealed multiple serpiginous burrows terminating in a brown triangular structure consistent with the “delta-wing jet” sign. In addition, whitish ovoid structures compatible with eggs/debris were observed, with focal ultraviolet-induced autofluorescence (Fig. 2), a pattern recently described as the “ball sign”.^3^ Direct microscopic examination of skin scrapings confirmed numerous live mites and eggs. During evaluation, the accompanying mother reported several weeks of nocturnal pruritus and had excoriated papules on the wrists and interdigital spaces, supporting household transmission.

**Fig. 2:**
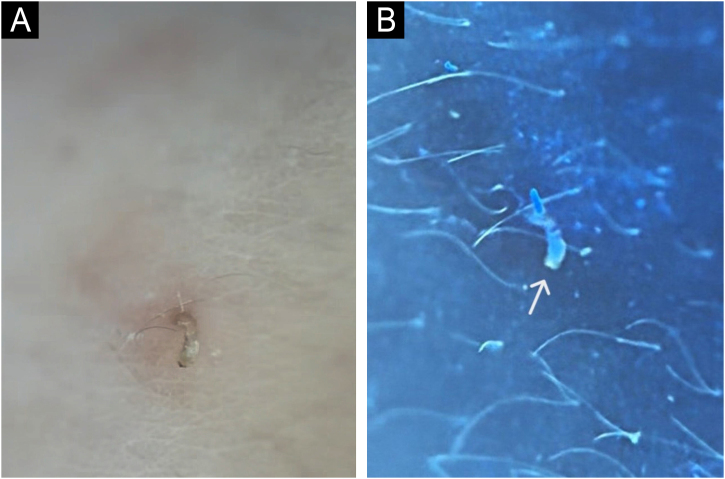
Dermoscopy and ultraviolet dermoscopy. (A) Dermoscopy showing a serpiginous burrow ending in a brown triangular structure (“delta-wing jet” sign). (B) Ultraviolet-induced fluorescence dermoscopy showing a focal blue-white, fluorescent ovoid structure (arrow), consistent with the “ball sign” (mite egg/debris).

Crusted scabies was diagnosed. Oral ivermectin use in children weighing < 15 kg remains off-label in many settings despite growing safety data in this weight group, ^4^ so we opted for intensive topical therapy. A case report has also described off-label high-dose oral ivermectin in an infant (1,200 μg/kg once weekly for 3-weeks) after poor response to topical therapy.^5^ The infant received repeated whole-body applications of 5% permethrin over two weeks, combined with measures to reduce the crust barrier (warm bathing, hydration, and gentle manual removal of crusts) and liberal emollients. All household contacts were treated simultaneously. Environmental control included hot laundering of clothing and bedding, and prolonged sun exposure of non-washable items. Pruritus and irritability improved within the first week, and near-complete clearance of erythroderma and crusting was achieved by week-3, with parallel resolution of symptoms in household contacts.

This case highlights three practical points. First, crusted scabies should remain on the differential diagnosis of infantile erythroderma, particularly when thick crusts and marked eosinophilia are present, even without known immunosuppression.^1^, ^2^ Second, dermoscopy is a rapid, noninvasive shortcut to confirmatory sampling and can increase diagnostic confidence in children; ultraviolet dermoscopy may provide additional visual cues.^3^ Third, because mite burden and transmissibility are extreme, simultaneous treatment of close contacts and environmental measures are essential to prevent rapid reinfestation and ongoing spread.^6^, ^7^

## ORCID ID

Márcio Fellipe Menezes Viana: 0009-0005-1663-9591

Renata Ferreira Magalhães: 0000-0001-9170-932X

Andréa Fernandes Eloy da Costa França: 0000-0003-1657-4570

## Patient consent statement

The authors obtained written consent from patients for their photographs and medical information to be published in print and online, and with the understanding that this information may be publicly available. Patient consent forms were not provided to the journal but are retained by the authors.

## Financial support

This study was funded exclusively by the authors, with no financial support from public or private institutions.

## Authors' contributions

Márcio Fellipe Menezes Viana: Conceptualization; investigation; data curation; writing-original draft; visualization.

Renata Ferreira Magalhães: Methodology; investigation; writing-review & editing.

Andréa Fernandes Eloy da Costa França: Supervision; conceptualization; validation; writing-review & editing.

All authors approved the final version of the manuscript and agree to be accountable for all aspects of the work.

## Research data availability

Does not apply.

## Conflicts of interest

None declared.

## References

[1] Segado Sánchez M., Lova Navarro M., Martínez Ortega F.J., Parra García J.J., López Martínez D., Sánchez-Pedreño Guillén P. (2024). Plantar keratoderma-like crusted scabies in an immunocompetent infant after topical steroids. Pediatr Dermatol..

[2] Tolkachjov S.N., Davis M.D.P., Yiannias J.A. (2018). Crusted (Norwegian) scabies: nine-month course with iatrogenic immunosuppression. J Drugs Dermatol..

[3] Arriel K., Jabour T.B.F., Rubinho R., Yanase T.K., Rytenband F. (2025). The big and small ball sign: ultraviolet-induced fluorescence dermoscopy for the diagnosis of scabies. Rev Soc Bras Med Trop..

[4] Levy M., Martin L., Bursztejn A.-C., Chiaverini C., Miquel J., Mahé E. (2020). Ivermectin safety in infants and children under 15 kg treated for scabies: a multicentric observational study. Br J Dermatol..

[5] Bourkas A.N., Pope E. (2023). Oral ivermectin treatment for an infant with crusted scabies. CMAJ..

[6] Uzun S., Durdu M., Yürekli A., Mülayim M.K., Akyol M., Velipaşaoğlu S. (2024). Clinical practice guidelines for the diagnosis and treatment of scabies. Int J Dermatol..

[7] Romani L., Whitfeld M.J., Koroivueta J., Kama M., Wand H., Tikoduadua L. (2015). Mass drug administration for scabies control in a population with endemic disease. N Engl J Med..

